# Compound dietary fiber and high-grade protein diet improves glycemic control and ameliorates diabetes and its comorbidities through remodeling the gut microbiota in mice

**DOI:** 10.3389/fnut.2022.959703

**Published:** 2022-07-22

**Authors:** Yinhua Ni, Aqian Zheng, Yating Hu, Nianke Rong, Qianpeng Zhang, Wenmin Long, Song Yang, Sujie Nan, Liqian Zhang, Kexin Zhou, Tianxing Wu, Zhengwei Fu

**Affiliations:** ^1^College of Biotechnology and Bioengineering, Zhejiang University of Technology, Hangzhou, China; ^2^Polaris Health Life Science Research Center, Zhejiang University of Technology, Hangzhou, China; ^3^Department of Chemistry, Zhejiang University, Hangzhou, China

**Keywords:** dietary fiber, diabetes, glycemic control, insulin resistance, inflammation, gut microbiota

## Abstract

Dietary intervention with a low glycemic index and full nutritional support is emerging as an effective strategy for diabetes management. Here, we found that the treatment of a novel compound dietary fiber and high-grade protein diet (CFP) improved glycemic control and insulin resistance in streptozotocin-induced diabetic mice, with a similar effect to liraglutide. In addition, CFP treatment ameliorated diabetes-related metabolic syndromes, such as hyperlipidemia, hepatic lipid accumulation and adipogenesis, systemic inflammation, and diabetes-related kidney damage. These results were greatly associated with enhanced gut barrier function and altered gut microbiota composition and function, especially those bacteria, microbial functions, and metabolites related to amino acid metabolism. Importantly, no adverse effect of CFP was found in our study, and CFP exerted a wider arrange of protection against diabetes than liraglutide. Thereby, fortification with balanced dietary fiber and high-grade protein, like CFP, might be an effective strategy for the management and treatment of diabetes.

## Introduction

Type 2 diabetes (T2D), characterized by insulin resistance with loss of islets β cells function, is on the rise worldwide, with a global prevalence of 537 million adults in 2021, and this number will reach to 783 million by 2045 (1). The current treatment strategy for T2D mainly includes the administration of oral hypoglycemic agents like sulfonylureas, metformin, and thiazolidinediones, and injectable drugs like insulin and insulin secretagogues (2). Meanwhile, with the development of novel antidiabetic agents, including glucagon-like peptide-1 (GLP-1) receptor agonists, dipeptidyl peptidase-4 (DPP-4) inhibitors, and sodium-dependent glucose transporter-2 (SGLT-2) inhibitors, the therapeutic alternatives for T2D are increasing (3). However, the possibilities of severe adverse effects and the large heterogeneity of T2D patients might still limit the usage of these drugs (4). Lifestyle change, such as a high-calorie diet, is the major risk for the progression of obesity and T2D, making dietary manipulation, such as calorie restriction and diet structure change, become one major direction to ameliorate T2D. In addition to low-calorie intake and time-restricted feeding, dietary and supplementation interventions also contribute to the management and improvement of diabetes (5). Therefore, a diabetes-friendly diet with a low glycemic index and full nutritional support is emerging as an effective strategy for diabetes management and is increasingly accepted by diabetic patients.

The gut microbiota structure is alterable over time, depending on the environmental condition, availability of nutrition and health state of the host, and in turn, the changes in gut microbiota also modulate the physiological function of the host (6). Studies have revealed that low richness or diversity of gut microbiota correlated with metabolic markers and might increase the risk of obesity-associated complications, including diabetes (7). In addition, the composition of the microbiota is more susceptible to be affected by the disease condition. For example, the abundance of *Ruminococcus*, *Fusobacterium*, and *Blautia*, which relate to inflammation and tumor progression in the gastrointestinal tract, is increased in the diabetic population, while the abundance of *Bifidobacterium*, *Bacteroides*, and *Akkermansia* is negatively associated with diabetes (8). Moreover, butyrate-producing bacteria, such as *Roseburia*, *Subdoligranulum*, and *Faecalibacterium*, are decreased in T2D individuals (9). Therefore, the current pharmacological therapies start to combine the microbiota-modification effect during the treatment of diabetes. For instance, the anti-hyperglycemic effect of metformin was mediated by modulating gut microbiota to inhibit intestinal farnesoid X receptor (FXR) signaling (10). In addition, a clinical trial demonstrated that liraglutide-induced gut microbial structural alteration was associated with hemoglobin A1c (HbA1c) and blood urea nitrogen in diabetes patients (11). In light of these, targeting the gut microbiota has become an emerging approach to improve systemic glucose metabolism in the diabetic population.

Dietary fiber is the indigestible part of plant foods and has been long appreciated as a healthy food. A high supplement of fibers, which is >25 g/day in women and >38 g/day in men, reduces insulin resistance and the risk of T2D by 20–30% (12). Although dietary fiber is indigestible for humans, it is an important energy source for the gut microbiota and can remodel gut microbiota structure by inducing a higher abundance of fiber-degrading microbiota (13, 14). Meta-analysis indicated that dietary fiber intake, especially fructans and galacto-oligosaccharides, improved the selective enrichment of *Bifidobacterium* and *Lactobacillus* (15). Insoluble fibers mainly impact satiety by decreasing hunger, prolonging satiation, and/or increasing peripheral satiety signals, but have no function in improving insulin sensitivity (16). On the other hand, soluble fibers are fermented by gut bacteria into short-chain fatty acids (SCFAs) and other secondary metabolites and then exert a broad range of health-promoting effects. A randomized controlled trial suggested that soluble dietary fiber delayed gastric emptying in diabetic persons, which was associated with the decrease of postprandial glycemic and insulin (17). Therefore, the combination of soluble and insoluble fibers and fiber types all matter to an effective of fiber-enriched diet.

Soybean protein and whey protein, two common high-grade proteins, have considerable effects on metabolic regulation and modulation, such as cholesterol-lowering, triglyceride-lowering, inhibition of fatty acid synthase, anti-obesity, and antidiabetic effects (18, 19). Soybean protein isolate and whey protein concentrate, the products of purified and concentrated of soybean protein and whey protein, are often used as food supplements.

In the present study, we proposed and examined the comprehensive effects of a novel compound dietary fiber and high-grade protein diet (CFP), which provide both the soluble and insoluble fibers, soybean protein, whey protein, and other nutrients with full nutritional support, such as minerals and vitamins, on the management of diabetes and its comorbidities using a streptozotocin (STZ)-induced diabetic mouse model. First, the roles of CFP on glycemic control, insulin resistance, lipid metabolism, and tissue inflammation were determined by comparisons with liraglutide. Next, the impacts of CFP on gut barrier function and microbiota were investigated. Finally, the effects of CFP on plasma metabolomics and the potential mechanism were clarified.

## Materials and methods

### Material and dosage information

Compound dietary fiber and high-grade protein diet (CFP), which constituted 45.5% fibers, 24.5% carbohydrate, 19.2% proteins, 3.6% lipids, and essential vitamins and minerals, was provided by Shanghai Polaris Health Co., Ltd, Shanghai, China. Dietary fiber included 84% of soluble fiber originated from fibersol-2, inulin, fructooligosaccharides, polydextrose, β-glucans, and raffinose, and 16% of insoluble fiber, mainly oat fiber. The high-grade protein was mostly soy protein isolate and whey protein concentrate. Detailed nutrition facts label is shown in Supplementary Table 1. The recommended dose of CFP for a 60 kg adult is 50 g/day, and the converted amount for a 20 g mouse was 0.2 g/day based on a conversion factor of 12.4 folds as described previously (20), which was the low dose of CFP treatment in the present study.

### Animal experimental design

Seven-week-old male C57BL/6J mice were purchased from China National Laboratory Animal Resource Center (Shanghai, China) and housed in conventional cages at 22°C under 12-h light/dark cycle. After 1 week of acclimatization, 70 mice were first fed with normal chow (NC, P1101 F-25, Salcom Co., Ltd., Shanghai, China, *n* = 10) or high fat diet (HFD, Research Diets Inc., D12492, New Brunswick, NJ, United States, *n* = 60) for 4 weeks. The diabetes was induced by an intraperitoneal injection STZ (40 mg/kg BW, Sigma-Aldrich, St. Louis, MO, United States) in HFD-fed mice for 5 days, and the NC-fed mice received an equal amount of sodium citrate buffer injection as described previously (21). The mice with fasting blood glucose ≥11.1 mmol/L after STZ injection were considered diabetic mice and selected for subsequent study.

To investigate the effect of CFP on diabetes and its complications, NC-fed mice were used as the control group, and body weight- and blood glucose-matched diabetic mice were divided into five groups and fed for 12 weeks as follows (Supplementary Figure 1A): (1) Diabetes group (Dia), HFD feeding and saline injection, *n* = 10; (2) Low-dose CFP group (Low), HFD feeding with 0.2 g/mouse/day CFP intake and saline injection, *n* = 10; (3) Medium-dose CFP group (Med), HFD feeding with 0.4 g/mouse/day CFP intake and saline injection, *n* = 10; (4) High-dose CFP group: HFD feeding with 0.6 g/mouse/day CFP intake and saline injection, *n* = 10; (5) Liraglutide group (Lira), HFD feeding and subcutaneous injection of liraglutide (AdooQ BioScience, Irvine, CA, United States, 0.2 mg/kg BW), *n* = 10. The corresponding amount of CFP was fed to mice during the first 2–4 h of the dark phase, and then the HFD was fed after they consumed all CFP every day. Due to the appetite-inhibiting effect of liraglutide, all diabetic mice pair-fed with the same calorie as the liraglutide-treated mice. The food and caloric intake were measured daily. Fed blood glucose was measured every week, and the fasting blood glucose was measured at baseline, 8 and 12 weeks after CFP treatment.

An oral glucose tolerance test (OGTT) was conducted after 8 weeks of treatment by oral gavage of glucose (0.5 g/kg BW) after 16 h of fasting. After 2 weeks of recovery, an insulin tolerance test (ITT) was performed after 12 h of fasting by intraperitoneal insulin injection (0.5 U/kg BW). Blood glucose was measured at the indicated time points. After 12 weeks of treatment, mice were sacrificed by cervical dislocation, blood and tissues, including the liver, fat, muscle, kidney, heart, and colon, were collected for further analyses. All experiments were approved by the Laboratory Animals Ethical Committee of the Zhejiang University of Technology and followed the NIH guide for laboratory animals (NIH Publication No. 85–23, revised 1996) for the care and use of animals.

### Biochemical analyses

Hepatic and plasma triglycerides (TG), total cholesterol (TC), and non-esterified fatty acids (NEFA) levels were measured by commercial kits from Fujifilm Wako Pure Chemical Corporation (Osaka, Japan). Plasma aspartate aminotransferase (AST), alanine transaminase (ALT), creatinine (CRE), high-density lipoprotein (HDL), low-density lipoprotein (LDL) were measured using commercial kits (Jiancheng Institute of Biotechnology Co., Ltd., Nanjing, China). Plasma insulin, lipopolysaccharide (LPS), and D-lactate (D-Lac) were measured using ELISA kits (Cusabio Technology Co., Ltd., Wuhan, China) according to the manufacturer’s instructions as described previously (22, 23). Homeostatic model assessment-insulin resistance (HOMA-IR) was calculated using the following equation: fasting glucose (mmol/L) × fasting insulin (mIU/L)/22.5.

### RNA extraction and quantitative real-time PCR

Total RNA of tissues was extracted using Biozol reagent (Biomiga Biochemicals, San Diego, CA, United States) according to the manufacturer’s instructions. cDNA was synthesized using the Goldenstar™ RT6 cDNA Synthesis Kit (Tsingke Co., Ltd., Beijing, China). Quantitative real-time PCR (qPCR) was performed on a CFX Connect Optics Module (Bio-Rad, Hercules, CA, United States) using Fast qPCR Mix (SYBR Green, Tsingke Co., Ltd., Beijing, China) as described previously (22). The primer sequences are shown in Supplementary Table 2.

### Immunoblots

Tissues were homogenized in radioimmunoprecipitation (RIPA) lysis buffer (Millipore, Billerica, MA, United States) with protease and phosphatase inhibitors (Roche Diagnostics). The primary antibodies used were anti-phospho-Ser473 serine/threonine protein kinase Akt (p-Akt) (#4060), anti-Akt (#9272), anti-phospho-nuclear factor kappa light chain-enhancer of activated B cells (NF-κB) p65 (Ser536) (#3033), anti-NF-κB (#8242), anti-phospho-p38 mitogen-activated protein kinases (p-p38 MAPK) (#9211), anti-p38 MAPK (#8690), anti-phospho-stress-activated protein kinase-c-Jun N-terminal kinase (p-SAPK/JNK) (#9255), anti-JNK (#9252), anti-phospho-extracellular signal-regulated kinases (p-ERK1/2) (#9101), anti-ERK1/2 (#4695) (Cell Signaling Technology, Danvers, MA, United States), and β-Actin (EM-21002, HuaBio, Hangzhou, China).

### Histopathological analyses

Paraffin-embedded liver and eWAT sections were stained with hematoxylin and eosin (H&E) and immunohistochemically stained for F4/80 (GB11027, Wuhan Servicebio Technology Co., Ltd., Wuhan, China), colonic sections were stained with H&E and Alcian blue-periodic acid Schiff (AB-PAS), kidney sections were stained with H&E, PAS, and Masson, and pancreatic sections were immunohistochemically stained for insulin (GB13121), glucagon (GB11097, Wuhan Servicebio Technology Co., Ltd., Wuhan, China) as described previously (22).

### 16S rRNA gene sequencing and data analysis

Total genomic DNA from cecal contents samples were extracted using the OMEGA Soil DNA Kit (M5635-02) (Omega Bio-Tek, Norcross, GA, United States). The quantity and quality of extracted DNAs were measured using a NanoDrop NC2000 spectrophotometer (Thermo Fisher Scientific, Waltham, MA, United States) and agarose gel electrophoresis, respectively. The 16S rRNA gene (V3–V4 region) was amplified with the forward primer 338F (5′-ACTCCTACGGGAGGCAGCA-3′) and the reverse primer 806R (5′-GGACTACHVGGGTWTCTAAT-3′). The PCR components contained 5 μL of buffer (5×), 0.25 μL of Fast pfu DNA Polymerase (5 U/μL), 2 μL (2.5 mM) of dNTPs, 1 μL (10 μM) of each Forward and Reverse primer, 1 μL of DNA Template, and 14.75 μL of ddH_2_O. Thermal cycling consisted of initial denaturation at 98°C for 5 min, followed by 25 cycles consisting of denaturation at 98°C for 30 s, annealing at 53°C for 30 s, and extension at 72°C for 45 s, with a final extension of 5 min at 72°C. The composition of the gut microbiome was determined by the Illumina NovaSeq platform at Personal Biotechnology (Shanghai, China). The raw reads of 16S rRNA gene sequences were analyzed using QIIME2 bioinformatic analysis as previously described (22). The sequence data were demultiplexed using the demux plugin followed by primers cutting with cutadapt plugin. High-quality sequences were clustered into operational taxonomic units (OTUs) at 97% cutoff by Vsearch (v2.13.4_linux_x86_64) (24), and the taxonomy was classified with Silva Database. The number of high-quality reads was more than 55,000 for each sample. Sequence data analyses were mainly performed using QIIME2 and R packages (v3.2.0).

### Non-target metabolomics analysis

Plasma samples were vortexed with methanol and centrifuged, and the supernatant was dried and resuspended in 2-chloro-l-phenylalanine with 80% methanol. Subsequent metabolite measurements were performed on a Vanquish UHPLC System (Thermo Fisher Scientific, Waltham, MA, United States). The mass spectrometric detection of metabolites was performed on Orbitrap Exploris 120 (Thermo Fisher Scientific, Waltham, MA, United States) with an ESI ion source. Simultaneous MS1 and MS/MS (Full MS-ddMS2 mode, data-dependent MS/MS) acquisition was used. All multivariate data analyses were mainly performed using R packages (v3.2.0), and differential metabolites were subjected to pathway analysis by MetaboAnalyst, which combines results from powerful pathway enrichment analysis with the pathway topology analysis. The identified metabolites in metabolomics were then mapped to the KEGG pathway for biological interpretation of higher-level systemic functions.

### Statistical analysis

All data were expressed as means ± SEM (mean ± standard error of the mean). The differences between the mean values were assessed using one-way ANOVA analysis, followed by the Student–Newman–Keuls test. *P*-values < 0.05 or 0.01 were considered statistically significant.

## Results

### Compound dietary fiber and high-grade protein diet administration lowers the blood glucose levels and improves insulin sensitivity in diabetic mice

**FIGURE 1:**
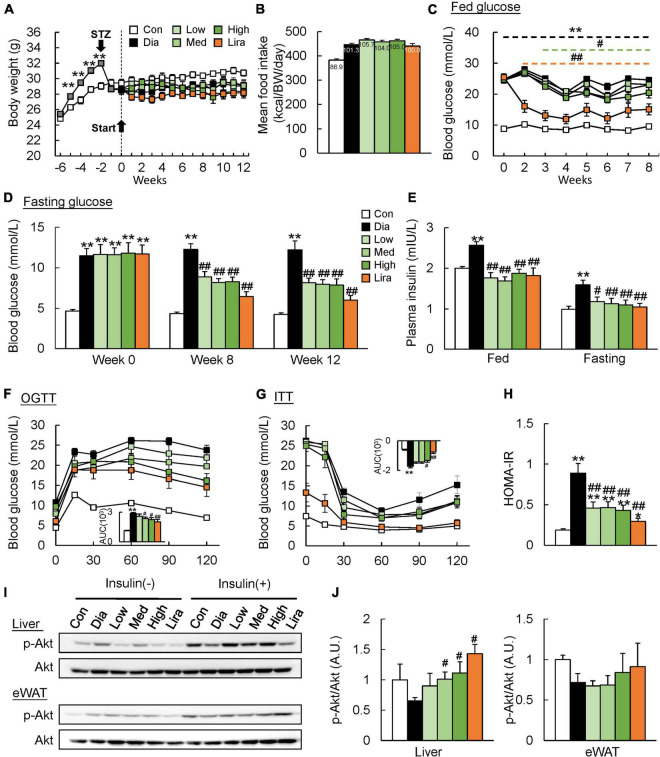
CFP administration improves glucose tolerance and insulin sensitivity. **(A)** Body weight. **(B)** Daily food intake. **(C)** Blood glucose levels at fed state. **(D)** Fasting blood glucose levels at the baseline and after 8 and 12 weeks of treatment. **(E)** Fed and fasting plasma insulin levels. **(F)** Oral glucose tolerance test (OGTT). AUC, area under the curve. **(G)** Insulin tolerance test (ITT). **(H)** HOMA-IR index. **(I,J)** Immunoblots and quantification data of p-Akt levels in the liver and eWAT. Data are presented as the means ± SEM, *P < 0.05, **P < 0.01 vs. control; ^#^*P* < 0.05, ^##^*P* < 0.01 vs. Dia.

The body weight of HFD-fed mice increased significantly from the second week and decreased to a level lower than control mice after STZ injection (Figure 1A). Meanwhile, the body weight of diabetic mice was not affected either by the different doses of CFP or liraglutide treatment (Figure 1A and Table 1). The daily calorie intake of diabetic mice was 14.4% higher than control mice, while no significant difference in both the total and daily calorie intake was found among CFP-treated and liraglutide-treated mice (Figure 1B and Supplementary Figure 1B). High dose CFP treatment started to lower the fed blood glucose level from the third week, and an earlier and more robust blood glucose-lowering effect was found in liraglutide-treated mice (Figure 1C). The fasting blood glucose level in diabetic mice was sustained higher than in control mice during the whole experiment process (Figure 1D). Consistently, though no difference was found at the baseline, CFP treatment significantly reduced the fasting blood glucose level after 8 and 12 weeks of treatment, leading the levels close to the liraglutide-treated mice, and no significant difference was found among the three doses of CFP-treated groups (Figure 1D). In addition, the plasma insulin levels both at the fed and fasting states were markedly decreased by both CFP and liraglutide treatment (Figure 1E). Moreover, glucose intolerance and insulin resistance caused by diabetes were ameliorated by CFP and liraglutide as assessed by OGTT, ITT, and HOMA-IR index, and the effect tended to be stronger in liraglutide-treated group (Figures 1F–H). These results were associated with significantly and slightly enhanced insulin signaling in the liver and eWAT of CFP- and liraglutide-treated mice (Figures 1I,J). Furthermore, the immunofluorescence data revealed that the number and size of pancreatic islets were markedly decreased in the diabetic mice, while either CFP or liraglutide had little recovery effect on the pancreatic damage (Supplementary Figure 1C).

**TABLE 1:** Effects of CFP on metabolic parameters of type 2 diabetes mice.

Parameters	Con	Dia	Low	Med	High	Lira
Body weight (g)	31.63 ± 0.39	29.54 ± 0.35**	29.35 ± 0.64**	29.36 ± 0.55**	28.67 ± 0.86**	29.0 ± 0.50**
Liver weight (g)	1.13 ± 0.03	1.22 ± 0.04	1.10 ± 0.05	1.07 ± 0.03^##^	1.04 ± 0.03*^##^	0.92 ± 0.03**^##^
Epididymal fat (g)	0.49 ± 0.05	0.83 ± 0.10**	0.71 ± 0.11	0.67 ± 0.07*	0.49 ± 0.09^#^	0.64 ± 0.04*
Kidney weight (g)	0.32 ± 0.01	0.34 ± 0.01	0.33 ± 0.01	0.32 ± 0.01	0.30 ± 0.01^#^	0.30 ± 0.01^##^
Quadriceps muscle (g)	0.35 ± 0.01	0.33 ± 0.02	0.31 ± 0.01*	0.32 ± 0.01	0.33 ± 0.01	0.31 ± 0.01*
Gastrocnemius muscle (g)	0.28 ± 0.01	0.26 ± 0.01	0.27 ± 0.01	0.26 ± 0.01	0.25 ± 0.01*	0.27 ± 0.01
Tibialis anterior muscle (g)	0.15 ± 0.00	0.13 ± 0.00**	0.12 ± 0.01*	0.13 ± 0.01*	0.12 ± 0.01**	0.13 ± 0.00*
Soleus muscle (g)	0.02 ± 0.00	0.02 ± 0.00	0.01 ± 0.00	0.02 ± 0.00	0.01 ± 0.00	0.02 ± 0.00
Plasma AST (U/L)	7.76 ± 1.20	18.04 ± 2.68**	10.83 ± 0.47*^#^	9.37 ± 0.90^##^	5.93 ± 0.61^##^	7.74 ± 0.89^##^
Plasma ALT (U/L)	4.20 ± 0.41	37.14 ± 5.58**	15.22 ± 1.94**^##^	8.98 ± 1.23**^##^	8.92 ± 1.42**^##^	8.38 ± 2.49^##^
Plasma TG (mg/dL)	64.89 ± 7.51	88.53 ± 3.52*	74.24 ± 4.19^#^	74.61 ± 4.04^#^	66.60 ± 7.28^#^	82.17 ± 8.25
Plasma TC (mg/dL)	84.84 ± 2.40	130.74 ± 6.61**	113.02 ± 4.94**^#^	109.83 ± 5.26**^#^	98.35 ± 6.80^##^	129.46 ± 6.29**
Plasma NEFA (mEq/L)	0.54 ± 0.06	0.77 ± 0.05*	0.58 ± 0.08	0.59 ± 0.09	0.53 ± 0.06^#^	0.62 ± 0.07
Plasma LDL (mmol/L)	0.44 ± 0.09	1.08 ± 0.09**	0.89 ± 0.16*	0.79 ± 0.06**^#^	0.68 ± 0.08^##^	0.58 ± 0.10^##^
Plasma HDL (mmol/L)	2.03 ± 0.09	1.60 ± 0.03**	1.34 ± 0.06**^##^	1.49 ± 0.17*	1.95 ± 0.13^#^	2.00 ± 0.14^#^
Plasma CRE (μmol/L)	14.53 ± 0.68	17.78 ± 0.83*	13.01 ± 1.24^#^	11.32 ± 1.31^##^	10.82 ± 0.55**^##^	13.00 ± 1.24^#^
Hepatic TG (mg/mg protein)	0.15 ± 0.02	0.18 ± 0.02**	0.13 ± 0.01^#^	0.12 ± 0.01^##^	0.10 ± 0.01*^##^	0.15 ± 0.02
Hepatic TC (mg/mg protein)	0.02 ± 0.00	0.04 ± 0.01*	0.02 ± 0.00^#^	0.02 ± 0.00^##^	0.02 ± 0.00^##^	0.02 ± 0.01^#^
Hepatic NEFA (μEq/mg protein)	0.06 ± 0.01	0.09 ± 0.01*	0.05 ± 0.01^##^	0.04 ± 0.01^##^	0.03 ± 0.00**^##^	0.05 ± 0.01^#^

Data are presented as mean ± SEM. *n* = 5–11. *P < 0.05, **P < 0.01 vs. Con; ^#^*P* < 0.05, ^##^*P* < 0.01 vs. Dia.

### Compound dietary fiber and high-grade protein diet treatment ameliorates metabolic disorders in diabetic mice

**FIGURE 2:**
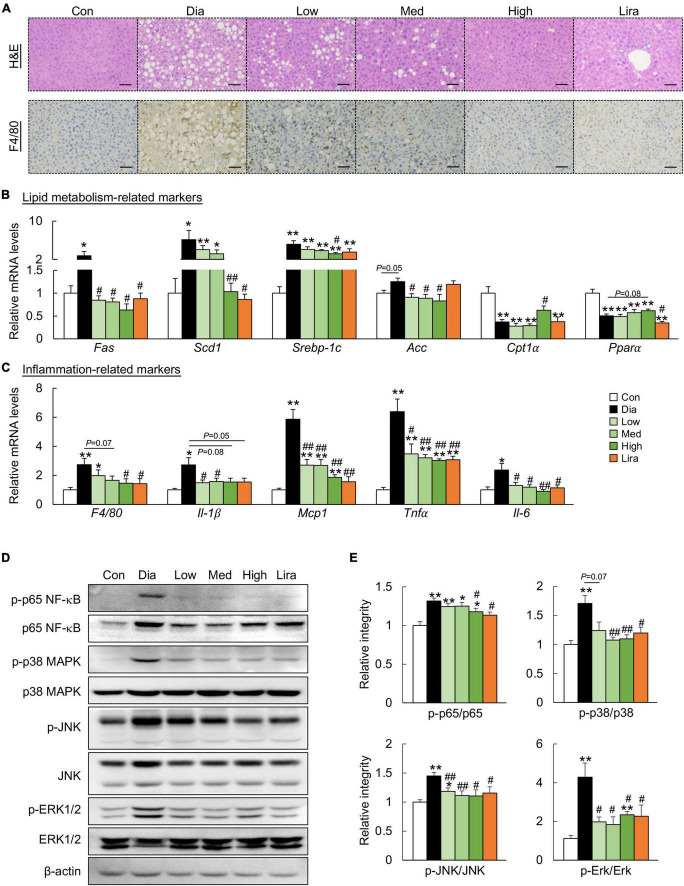
CFP administration ameliorates metabolic disorders in the liver. **(A)** H&E-staining and immunohistochemistry staining for F4/80 in the liver, Scale bar: 100 μm. **(B)** mRNA expression of the lipid metabolism-related markers in the liver. **(C)** mRNA expression of the inflammation-related markers in the liver. **(D)** Immunoblots of p-p65 NF-κB, p65 NF-κB, p-p38 MAPK, p38 MAPK, p-JNK, JNK, p-ERK1/2, and ERK1/2 in the liver. **(E)** Quantification data for p-p65 NF-κB, p65 NF-κB, p-p38 MAPK, p38 MAPK, p-JNK, JNK, p-ERK1/2, and ERK1/2 in the liver. Data are presented as the means ± SEM, *P < 0.05, **P < 0.01 vs. control; ^#^*P* < 0.05, ^##^*P* < 0.01 vs. Dia.

Though the body weight was not affected, high dose CFP and liraglutide treatment also caused a prominent decrease in liver weight and kidney weight (Table 1). The eWAT weight was only reduced by high dose CFP treatment, while the typical muscle weights remained unaffected in diabetic mice (Table 1). Plasma AST and ALT levels, the liver function markers, were increased significantly in diabetic mice, which were markedly and dose-dependently decreased by CFP and liraglutide treatment (Table 1). Additionally, diabetes and HFD-induced hyperlipidemia, including elevated plasma TG, TC, and NEFA levels, was significantly improved by CFP treatment, but not in liraglutide-treated group (Table 1). Histological analysis revealed larger and abundant lipid droplets in the liver of diabetic mice, and CFP and liraglutide treatment decreased lipid accumulation as determined by reduced hepatic TG, TC, and NEFA contents (Figure 2A and Table 1). Consistent with these findings, the mRNA expressions of lipogenic genes, such as fatty acid synthetase (*Fas*), stearoyl-CoA desaturase-1 (*Scd1*), sterol regulatory element binding protein-1c (*Srebp-1c*), and acetyl CoA carboxylase (*Acc*), were all significantly downregulated by CFP administration, and slightly by liraglutide (Figure 2B). In contrast, the mRNA expression of fatty acid oxidation-related markers, including carnitine palmitoyl transferase 1α (*Cpt1*α) and peroxisome proliferators-activated receptor α (*Ppar*α), was upregulated by CFP, but not liraglutide (Figure 2B).

Next, the role of CFP on hepatic inflammation was determined. F4/80 immunostaining indicated that CFP and liraglutide treatment reduced the activation of F4/80^+^ macrophages in the liver of diabetic mice (Figure 2A), which was further confirmed by the mRNA expression of *F4/80* (Figure 2C). These results were accompanied by decreased expression of proinflammatory cytokines and chemokines, such as interleukin (*Il*)-*1*β, monocyte chemotactic protein 1 (*Mcp1*), tumor necrosis factor α (*Tnf*α), and *Il-6* in the liver (Figure 2C). Moreover, the inflammatory pathway, characteristic by the phosphorylation of p65 NF-κB, p38 MAPK, JNK, and ERK, was activated in the liver of diabetic mice, which was attenuated by CFP and liraglutide (Figures 2D,E).

**FIGURE 3:**
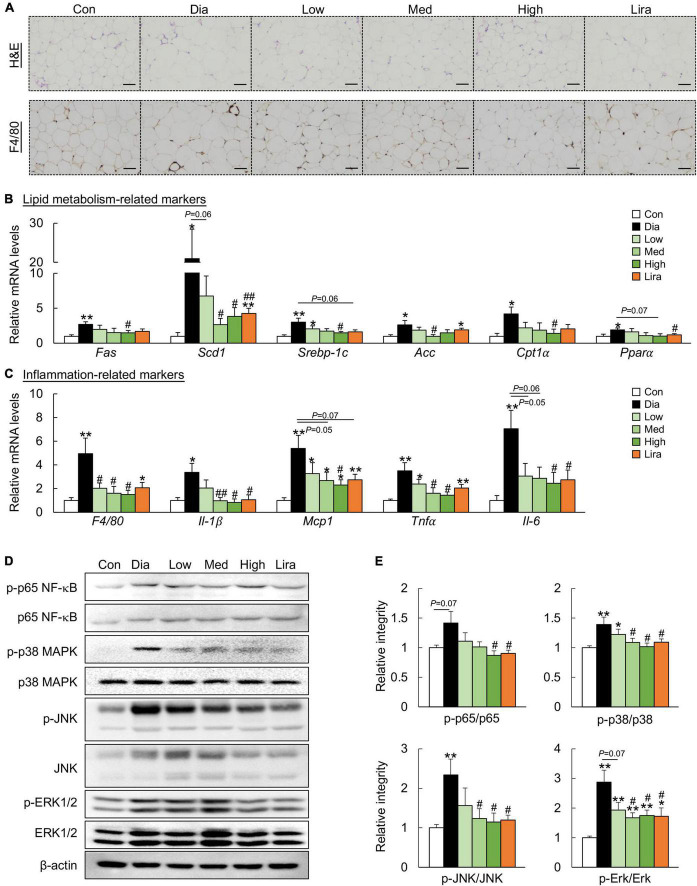
CFP administration ameliorates metabolic disorders in the eWAT. **(A)** H&E staining and immunohistochemistry staining for F4/80 in eWAT, Scale bar: 100 μm. **(B)** mRNA expression of the adipogenic genes in the eWAT. **(C)** mRNA expression of the inflammatory cytokines in the eWAT. **(D)** Immunoblots of p-p65 NF-κB, p-p38 MAPK, p-JNK, and p-Erk1/2 in the eWAT. **(E)** Quantification data for p-p65 NF-κB, p-p38 MAPK, p-JNK, and p-ERK1/2 in the eWAT. Data are presented as the means ± SEM, *P < 0.05, **P < 0.01 vs. control; ^#^*P* < 0.05, ^##^*P* < 0.01 vs. Dia.

Similarly, CFP treatment attenuated adiposity and downregulated the expression of the adipogenic genes in the eWAT of diabetic mice (Figures 3A,B). In addition, CFP treatment also ameliorated adipose tissue inflammation, as determined by F4/80 immunostaining, mRNA expression of inflammatory markers, and protein markers of the inflammatory pathway (Figures 3A,C–E). The overall anti-inflammatory effect of high dose CFP was similar to that of liraglutide.

### Compound dietary fiber and high-grade protein diet reduces the risk of diabetic comorbidities

We next investigated the effect of CFP on diabetic nephropathy, a common complication of diabetes. The plasma creatinine, as one kidney injury marker, increased significantly in diabetic mice, and CFP treatment resulted in a robust decrease in the plasma creatinine level (Table 1). The effect of liraglutide on creatinine was similar to that of low-dose CFP treatment (Table 1). Histological analyses also revealed that CFP and liraglutide treatment reduced lipid accumulation, improved the morphology of the renal glomerulus and mesangial cells, as well as attenuated renal fibrosis (Supplementary Figure 2A). These results were further verified by the downregulation of the expression of inflammatory cytokines and chemokines, and fibrogenic genes, such as α smooth muscle actin (α*Sma*), collagen type I alpha 1 chain (*Col1a1*), and transforming growth factor β (*Tgf-*β), in the kidney of CFP- and liraglutide-treated mice (Supplementary Figures 2B,C). On the other hand, elevated plasma LDL and lowered HDL levels caused by diabetes were reversed by CFP and liraglutide (Table 1). However, the mRNA expression of inflammatory and heart failure markers remained unchanged in the heart of diabetic mice (Supplementary Figures 2D,E).

### Compound dietary fiber and high-grade protein diet protects the gut barrier function of diabetic mice

**FIGURE 4:**
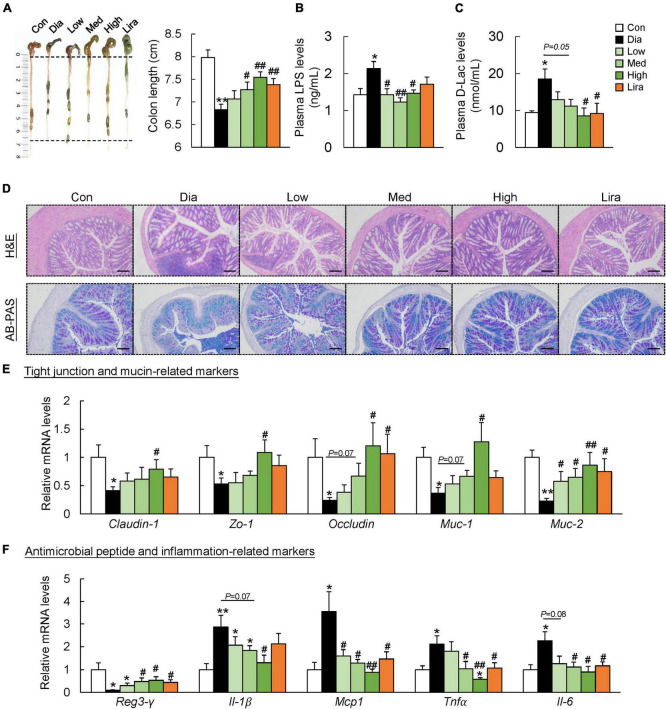
CFP administration improves the gut barrier function. **(A)** Representative pictures of the colons and colon length. **(B)** Plasma LPS levels. **(C)** Plasma D-Lac levels. **(D)** H&E and AB-PAS staining of the colon, Scale bar: 100 μm. **(E)** mRNA expression of the tight junction- and mucin-related markers in the colon. **(F)** mRNA expression of the antimicrobial peptide- and inflammation-related markers in the colon. Data are presented as the means ± SEM, *P < 0.05, **P < 0.01 vs. control; ^#^*P* < 0.05, ^##^*P* < 0.01 vs. Dia.

We next investigated the effects of CFP on gut barrier function. The colon length was shorter in the diabetic mice than in the control mice, and it was restored by CFP treatment in a dose-dependent manner (Figure 4A). In addition, plasma LPS and D-lactate levels, indicators for intestinal permeability, increased significantly in the diabetic mice, which were reduced by CFP treatment (Figures 4B,C). Liraglutide had a similar effect on these markers (Figures 4A–C). Moreover, CFP administration alleviated inflammatory cell infiltration in the colonic tissue and increased the mucus layer as determined by H&E and AB-PAS staining (Figure 4D). Consistent with the histological results, the mRNA levels of tight junction and mucin-related markers, including *Claudin-1*, zonula occludens 1 (*Zo-1*), *Occludin*, mucin (*Muc*)-*1*, and *Muc-2*, in the colon were all upregulated by CFP (Figure 4E). On the other hand, higher expression of regenerating islet-derived protein 3-γ (*Reg3-* γ) and lower expression of inflammatory markers was found in the colon of CFP-treated mice (Figure 4F). The effect of liraglutide on the tight junction- and mucin-related markers tended to be weaker than CFP, while the anti-inflammatory effect was similar to CFP (Figures 4E,F).

### Compound dietary fiber and high-grade protein diet remodels the composition and function of gut microbiota

**FIGURE 5:**
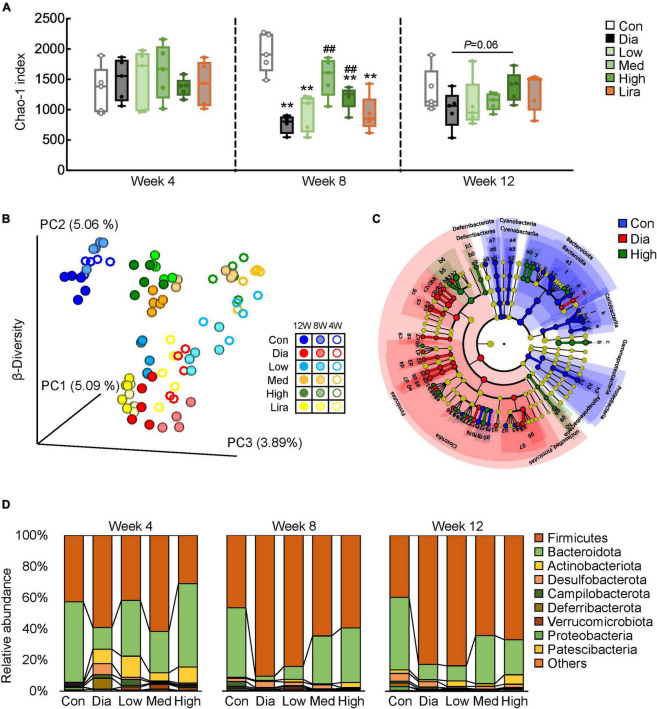
CFP administration remodels the composition of gut microbiota. **(A)** α-Diversity of the gut microbial communities in the feces after 4, 8, and 12 weeks of treatment. Data are presented as the means ± SEM, *P < 0.05, **P < 0.01 vs. control; ^#^*P* < 0.05, ^##^*P* < 0.01 vs. Dia. **(B)** β-Diversity of the gut microbial communities in the feces. **(C)** Cladogram generated from LEfSe analysis between the control, diabetic, and high-dose CFP-treated mice. **(D)** Relative abundance of microbiota at the phylum level.

To determine the potential involvement of gut microbiota in CFP-mediated amelioration of diabetes and its complications, 16S rRNA sequencing of fecal microbiota was performed after 4, 8, and 12 weeks of treatment. The α-diversity, estimated by the Chao-1 index, was lower in the diabetic mice and increased by CFP after 8 weeks of treatment, which was further verified by the overall changes in Chao-1 index (Figure 5A). Principal coordinate analysis (PCoA) revealed clear discrimination in the β-diversity between control and diabetic mice, diabetic and different dose CFP-treated mice, leading to the spatial position of CFP-treated groups close to that of control mice (Figure 5B). On the other hand, liraglutide had little effect on the α-diversity and β-diversity of the fecal microbiota (Figures 5A,B), and thus the subsequent analyses were mainly focused on CFP treatment. Next, the taxonomic cladogram obtained by LEfSe analysis demonstrated marked differences in the taxa among the control, diabetic and high-dose CFP-treated mice (Figure 5C). The overall composition of gut microbiota was further analyzed at the phylum and genus levels. The abundance of *Firmicutes* increased while *Bacteroidetes* decreased in diabetic mice from week 4, and it lasted until the end of the study, which was partly reversed by different doses of CFP treatment (Figure 5D). Among the top 40 abundant bacterial taxa, CFP treatment resulted in clear enrichment of *Dubosiella*, *Parasutterella*, *Ruminococcaceae*, *Muribaculum*, *Allobaculum*, and *Bifidobacterium*, and mostly reversed the bacterial change caused by diabetes (Supplementary Figure 3).

Next, PICRUSt2 analysis was performed to predict the functional profiles of microbial communities, and the results were classified by METACYC categories. There were a total of 33 functions that were significantly altered by diabetes or high-dose CFP treatment (Supplementary Figure 4A). Among them, the functions related to the generation of precursor metabolite and energy, C1 compound utilization and assimilation, carbohydrate biosynthesis, amino acid biosynthesis, pyrimidine nucleotide biosynthesis, cell structure biosynthesis, and cofactor, prosthetic group, electron carrier, and vitamin biosynthesis were decreased. In contrast, the functions related to pyrimidine nucleotide degradation was enhanced in diabetic mice (Supplementary Figure 4A). The abnormal alterations in these functions were mostly recovered by high-dose CFP treatment (Supplementary Figure 4A).

**FIGURE 6:**
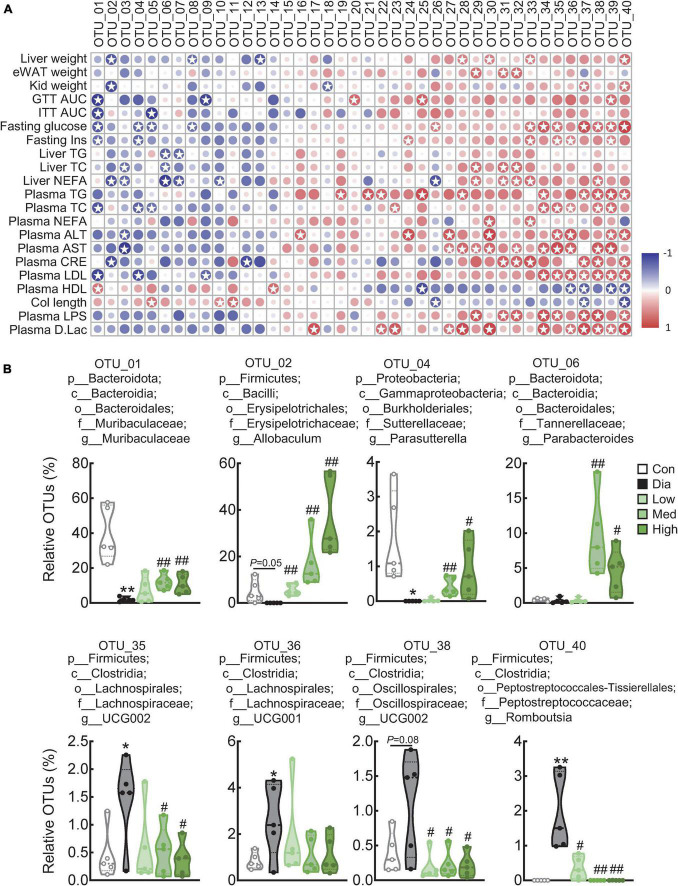
Correlation analysis of top 40 genera with diabetes-related parameters. **(A)** Correlation analysis of top 40 genera with diabetes-related parameters between the control, diabetic, and high-dose CFP-treated mice. Rows correspond to diabetes-related parameters, and columns correspond to the specific genus. Red and blue colors denote positive and negative associations, respectively. The intensity of the colors represents the degree of association between the abundance of bacteria and host parameters assessed by Pearson correlation analysis. Stars means *P* < 0.05. **(B)** Relative abundances of *Muribaculaceae*, *Allobaculum*, *Parasutterella*, *Parabacteroides*, *Romboutsia*, *Oscillospiraceae UCG 002*, *Lachnospiraceae UCG 001*, and *Lachnospiraceae UCG 002* in the feces. Data are presented as the means ± SEM, *P < 0.05, **P < 0.01 vs. control; ^#^*P* < 0.05, ^##^*P* < 0.01 vs. Dia.

Pearson correlation analysis was performed to identify the bacteria responsible for the effects of CFP in diabetes, and we found that several genera displayed strong positive or negative correlations with blood glucose and insulin resistance-related markers and plasma parameters (Figure 6A and Supplementary Table 3). Specifically, *Muribaculaceae*, *Allobaculum*, *Dubosiella*, *Prevotellaceae UCG 001*, *Parasutterella*, *Parabacteroides*, and *Bifidobacterium* were negatively correlated, while *Staphylococcus*, *Ruminococcus*, *Corynebacterium*, *Aerococcus*, *Lactobacillus*, *Clostridia vadin BB60 group*, *Lachnospiraceae UCG 002*, *Lachnospiraceae UCG 001*, *Clostridium sensu stricto 1*, *Oscillospiraceae UCG 002*, *Oscillospiraceae UCG 001*, and *Romboutsia* were positively correlated with these hyperglycemic and hyperlipidemic parameters (Figure 6A and Supplementary Table 3). Consistent with these findings, the relative abundance of *Muribaculaceae*, *Allobaculum*, *Parasutterella*, and *Parabacteroides* decreased significantly in diabetic mice, and those bacteria were dose-dependently enriched by CFP (Figure 6B). In contrast, the abundance of *Lachnospiraceae UCG 001* and *UCG 002*, *Oscillospiraceae UCG 002*, and *Romboutsia* increased in diabetes and was reduced by CFP treatment (Figure 6B). Moreover, the alteration of these bacterial genera, especially those strongly associated with the diabetic parameters, were also correlated with the functions related to the generation of precursor metabolite and energy, C1 compound utilization and assimilation, carbohydrate biosynthesis, amino acid biosynthesis (Supplementary Figure 4B).

### Compound dietary fiber and high-grade protein diet administration alters the plasma metabolome in diabetic mice

**FIGURE 7:**
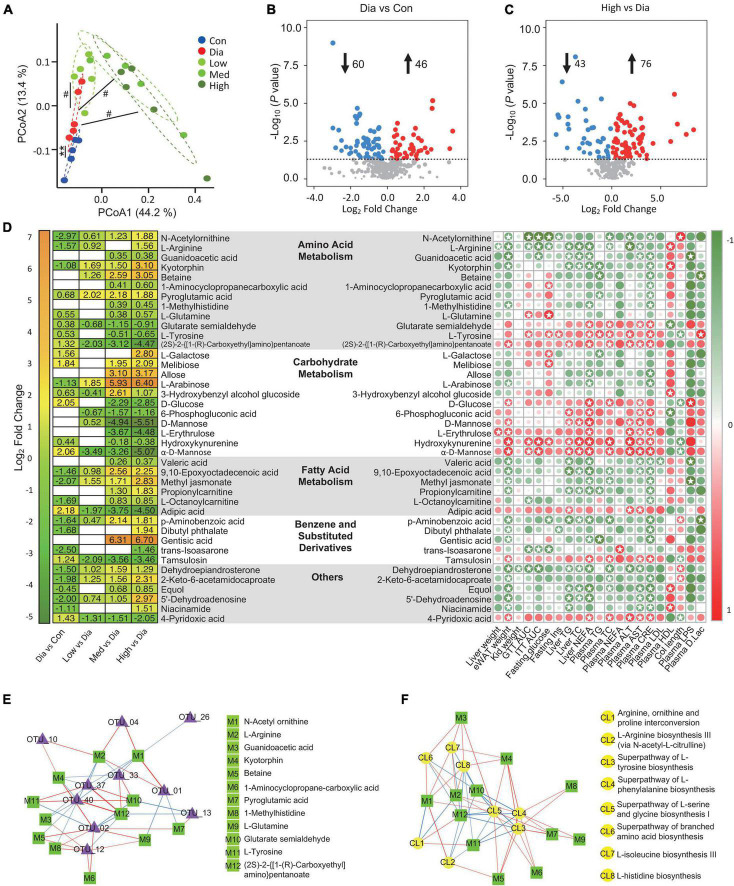
CFP administration alters the plasma metabolome. **(A)** Bray-Curtis Dissimilarity of MS/MS extracts from the experimental groups. ^**^P < 0.01 vs. control; ^#^*P* < 0.05 vs. Dia. **(B)** Volcano plot of differently expressed metabolites in the diabetic mice compared with the control ones. **(C)** Volcano plot of differently expressed metabolites in the high dose CFP-treated mice compared with the diabetic ones. **(D)** Statistic of differently expressed metabolites and correlation analysis with diabetes-related parameters. (Left) Heatmap of differently expressed metabolites from the experimental groups. (Right) Correlation analysis of metabolites with the physiological indexes. The intensity of the colors represents the degree of association between the levels of metabolites and host parameters assessed by Pearson correlation analysis. Stars means *P* < 0.05. **(E,F)** Network map depicting the relationships between metabolites and the composition **(E)** or the function **(F)** of gut microbiota. The red line means positive relationship, whereas the blue line means negative relationship.

To further investigate the metabolites that might be responsible for the effect of CFP, a metabolome analysis of plasma samples was performed. PCoA analysis revealed obvious differences in the plasma metabolite profiles of control, diabetic and CFP-treated mice (Figure 7A). Volcano plots further indicated that diabetes resulted in a decrease of 60 metabolites and an increase of 46 metabolites compared to the control mice (Figure 7B). In addition, high-dose CFP treatment caused 43 and 76 metabolites to be decreased and increased, respectively, compared to diabetic mice (Figure 7C). KEGG categories showed that these alterations in the levels of the metabolites were mainly related to the pathways involved in carbohydrate and amino acid metabolism, in addition to protein digestion and absorption, biosynthesis of plant secondary metabolites, ABC transporters, and biosynthesis of cofactors (Supplementary Figure 5).

To be specific, metabolites related to amino acid metaboli sms, such as N-acetylornithine, L-arginine, guanidoacetic acid, kyotorphin, betaine, 1-aminocyclopropanecarboxylic acid, pyroglutamic acid, 1-methylhistidine, and L-glutamine, were all increased by CFP treatment (Figure 7D). On the contrary, consistent with the blood glucose-lowering effect of CFP, metabolites associated with carbohydrate metabolism, including D-glucose, 6-phosphogluconic acid, D-mannose, L-erythrulose, hydroxykynurenine, and α-D-Mannose, were all decreased after the treatment of CFP (Figure 7D). In addition, the fatty acid metabolites were also enhanced by CFP, especially the short-chain fatty acid, valeric acid (Figure 7D). Moreover, benzene and substituted derivatives and other metabolites were also affected by CFP treatment (Figure 7D).

Pearson correlation analysis of these metabolites and physiological indexes revealed that specific kinds of metabolites displayed positive or negative correlations with diabetic phenotypes (Figure 7D). For instance, the above carbohydrate-related metabolites showed strong positive correlations with most diabetic parameters, while the increased amino acid metabolites were negatively correlated with these traits (Figure 7D). In addition, the tyrosine was positive correlations with diabetic phenotypes, and was decreased after CFP treatment (Figure 7D). Consistent with these findings, CFP reversed the changes in mRNA expression of arginine metabolism, tyrosine metabolism, and glycolysis in the liver of diabetes mice (Supplementary Figure 6).

On the other hand, we also found that the abundances of bacteria were found to be closely associated with these metabolites in the plasma (Supplementary Figure 7), and lots of them were also correlated with the functions related to amino acid biosynthesis (Supplementary Figure 4B). Next, the relationships between the top 10 bacterial genera related to the predicted function of amino acid biosynthesis of microbiota and the plasma level of amino acid metabolites were determined. Interestingly, the levels of N-acetylornithine, L-arginine, guanidoacetic acid, and kyotorphin were negatively correlated, while glutarate semialdehyde and (2S)-2-{[1-(R)-Carboxyethyl]amino}pentanoate were positively correlated with *Lactobacillus*, *Clostridium sensu stricto 1*, *Romboutsia* (Figure 7E). In addition, N-acetylornithine and L-arginine were also positively correlated with *Muribaculaceae* and *Parasutterella* (Figure 7E). Moreover, L-arginine, glutarate semialdehyde, and (2S)-2-{[1-(R)-Carboxyethyl]amino}pentanoate were strongly correlated with the function related to the superpathway of L-tyrosine, L-phenylalanine, L-serine and glycine, L-isoleucine, and L-histidine biosynthesis (Figure 7F).

## Discussion

A high-calorie diet is the main cause of the progression of obesity and T2D, making dietary manipulations, such as calorie restriction and diet structure change, become one major direction to ameliorate T2D, and among these manipulations high dietary fiber intake has long been proved to be especially effective in improving glycemic control, attenuating hyperinsulinemia and reducing inflammation in T2D patients (25, 26). Here, we found that CFP treatment significantly decreased hyperglycemia, hyperinsulinemia, and insulin resistance in diabetic mice, leading to effects close to those of liraglutide. Moreover, the impact of CFP on hyperlipidemia and hepatic steatosis was more potent than liraglutide, even though the anti-inflammatory effect in the liver, eWAT, and kidney was similar. On the other hand, clinical trials have demonstrated that liraglutide may lead to a series of adverse events, such as hypoglycemia, acute gallstone disease, acute cholecystitis, and the most common and the leading cause of the discontinuation of liraglutide, gastrointestinal disorders (27). The appetite-inhibiting effect of liraglutide was also found in our study, which was why pair-feeding was adopted to ensure the same amount of calorie intake. Therefore, compared with liraglutide, CFP exerted a similar glycemic control effect and a stronger lipid-lowering effect in the diabetic mice, suggesting that CFP might be one potent alternative of dietary intervention for the treatment of T2D.

The intestinal barrier function is mainly regulated by the gut microbiota-composed biological barrier and the physical barrier, which constitutes the luminal mucus layer, the gut epithelial layer, and the internal mucosal immune system (28). Numerous studies have demonstrated the fundamental role of the intestinal barrier in health and disease, and the dysfunction of the gut barrier is associated with a wide array of human diseases, including gastrointestinal and extra-intestinal disorders, such as obesity and diabetes (29). Dietary fiber deprivation or HFD-feeding will destroy the gut barrier by enriching mucus-degrading microbiota and decreasing the tight junction proteins (13, 23), leading to the release of LPS into circulation and contributing to tissue inflammation and insulin resistance (30). We found that CFP protected gut barrier function by alleviating endotoxemia and enhancing tight junction and mucin secretion, and these effects were stronger in high-dose CFP treated mice than those of liraglutide-treated ones. Therefore, different from the potent glycemic control and anti-inflammation effect of liraglutide, CFP might exert a wider range of protection against diabetes and its complications.

A growing body of evidence has clarified the critical roles of gut microbiota in the pathogenesis of metabolic diseases, including T2D, making the microbiota-targeted therapies, such as probiotics, prebiotics, and even fecal microbiota transplantation (FMT), translate from basic research into clinical studies and become one of the hottest areas of market application (31). Dietary fiber, the well-known prebiotic, has proven to effectively alleviate T2D both in animal and human studies (32). Previous studies have found that both insoluble fibers and soluble fibers effectively reduce blood glucose, while only soluble fibers improve insulin sensitivity, probably due to the short-chain fatty acids-promoting effect of soluble fibers, such as oat fiber and inulin (16). In addition, soluble and insoluble fibers regulate the microbiota profile differently. Moreover, the type, quality, and origin of food also shape the gut microbiota and impact the host-microbiota interactions. Therefore, a diet with high quality, good sources, and a balanced formation of soluble and insoluble fiber may be one potent strategy for microbiota-based dietary interventions for managing T2D, such as the CFP used in the current study. On the other hand, gut microbiota regulates the function of the pancreas, and dietary fibers are found to improve pancreatic beta-cell function and insulin secretion (33). However, our data suggested that neither CFP nor liraglutide affected the pancreatic function in diabetic mice, which might be caused by the STZ-induced severe damage to the pancreas.

Though several studies have suggested that liraglutide modulates the gut microbiota in animals and humans (11, 34), we found that liraglutide had little effect on the diversity of the microbiota. In contrast, CFP treatment resulted in robust alterations in the composition and function of microbiota, especially those significantly correlated with the diabetic parameters. Specifically, *Muribaculaceae* possesses several starch-degrading enzymes and is thereby involved in complex carbohydrate degradation (35). Studies have demonstrated that *Muribaculaceae* is negatively correlated with metabolic syndromes, such as diet-induced obesity, inflammatory bowel disease, and diabetes (35). Dietary fiber, such as inulin and resistant starch, could enrich the proportion of *Muribaculaceae* (36). *Allobaculum* plays multiple roles in regulating inflammation and lipid metabolism. For example, a recent study demonstrated that *Allobaculum* reduces colonic inflammation by producing 3-hydroxyoctadecadienoic acid, and the abundance of *Parasutterella* tended to increase in individuals consuming resistant potato starch, accompanied by a significant reduction of LDL levels. Oral administration of the strain of *Parabacteroides distasonis* alleviates obesity-related metabolic dysfunctions via the production of succinate and secondary bile acids (37). On the other hand, clinical data showed that the abundance of *Romboutsia* was positively correlated with body weight and serum lipid levels (38). Thus, the amelioration of T2D by CFP might be related to the enrichment of these beneficial bacteria and the reduction of harmful bacteria. However, the abundance of *Parasutterella* was decreased after inulin treatment, which was contrary to our results (39). The difference might be caused by the combination of different soluble and insoluble fibers in CFP. Further studies are required identify the specific strain of bacteria that responsible for the effect of CFP in diabetes.

Dietary fiber-mediated short-chain fatty acids production by microbiota has long been proved to be pivotal for ameliorating metabolic diseases, and consistently, we also found a significant increase in plasma valeric acid level. To further clarify and determine whether other metabolites except short-chain fatty acids are responsible for the effect of CFP, we focused on plasma metabolomics. We found that carbohydrate-related metabolites, especially those closely related to increased blood glucose levels, were all decreased by CFP. L-arabinose has been reported to regulate hyperglycemia by inhibiting hepatic gluconeogenesis through activating AMPK (40). Thus, with the observation of CFP-induced significant increase of L-arabinose, CFP exerted robust glycemic control properties through these two aspects. More importantly, our results revealed that the intake of soy protein isolate and whey protein concentrate in CFP might result in altered amino acid metabolism in the host and also lead to the change of amino acid metabolism-related functions in the microbial communities, which was further verified by the markedly altered amino acid-related metabolites in the plasma. Studies have indicated that soybean protein and whey protein showed considerable effects on metabolic regulation, such as lowering plasma lipid levels, reducing glycemia, enhancing the secretion of insulin and glucagon-like peptide-1 (18). Therefore, the antidiabetic effect of CFP might be also partly attributed to the intake of these high-grade proteins.

Regarding the amino acid-related metabolites, a recent study has found that the administration of N-acetylornithine, an antioxidative reagent for clinical diseases, ameliorated glucose metabolic disorder in HFD-fed mice partly through the protection of gut barrier function and the alleviation of gut dysbiosis (41). Soybean protein is a considerable source of L-arginine, and L-arginine has been regarded as a novel and effective therapy for obesity, diabetes, and metabolic syndrome. Kyotorphin is a unique biologically active neuropeptide (L-tyrosine-L-arginine), and it may exert an antidiabetic effect through its neuroprotective properties (42). In addition, a high serum level of betaine was associated with a low risk of T2D, and the supplementation of betaine improved insulin resistance in obese mice (43). Taken together, the increased levels of these metabolites by CFP might all contribute to the amelioration of diabetes found in the current study. On the other hand, a recent study revealed that elevated tyrosine level was associated with an increased risk of diabetic nephropathy in patients (44). p-Cresol is synthesized by intestinal anaerobic bacteria from aromatic amino acids, such as tyrosine and phenylalanine, and then sulfated into p-cresol sulfate (pCS), a prototype protein-bound uremic toxin that induces oxidative stress, inflammation, and fibrosis in the kidney proximal tubular cells (45). Therefore, the protection against kidney damage by CFP might be partly regulated by microbiota-mediated tyrosine metabolism. Further studies are required to clarify the mechanism of CFP and microbiota metabolites in diabetes and its comorbidities.

**FIGURE 8:**
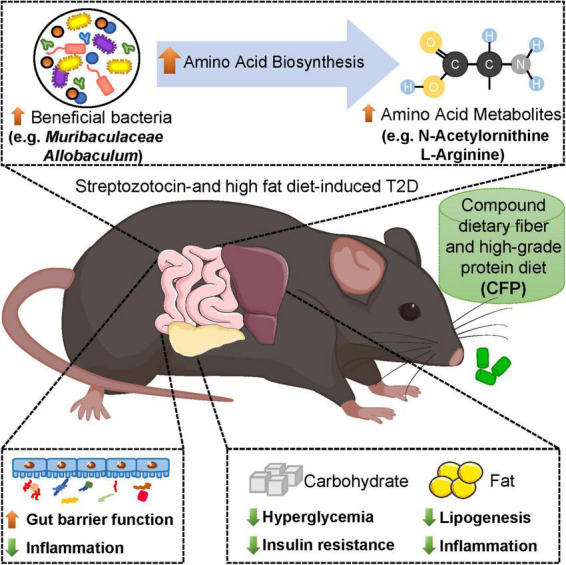
Schematic representation of the beneficial effects of CFP on diabetes. Compound dietary fiber and high-grade protein diet (CFP) supplementation significantly attenuated hyperglycemia, insulin resistance, lipid accumulation and inflammation in streptozotocin-induced type 2 diabetes (T2D) mice. Moreover, CFP treatment enhanced the gut barrier function and altered the composition and function of gut microbiota, especially those bacteria and metabolites closely correlated with diabetic parameters, and these bacterial functions and metabolites were mainly related to amino acid metabolism. Therefore, CFP ameliorated metabolic disorders of T2D, and might be an effective strategy for the management and treatment of diabetes.

In summary, our results suggested that CFP treatment improved hyperglycemia, hyperinsulinemia, and insulin resistance in diabetic mice, and these effects were similar to liraglutide (Figure 8). In addition, CFP significantly ameliorated metabolic disorders, including hyperlipidemia, liver dysfunction and hepatic steatosis, hepatic and adipose tissue inflammation, and diabetes-related comorbidities, such as diabetic nephropathy (Figure 8). Moreover, CFP treatment enhanced the gut barrier function and altered the composition and function of gut microbiota, especially those bacteria closely correlated with diabetic parameters, which may change the metabolites in the blood. These bacterial functions and metabolites were mainly related to amino acid metabolism (Figure 8). Importantly, unlike the adverse effect caused by liraglutide, such as appetite inhibition, no adverse effect of CFP was found in our study, and CFP exerted a wider arrange of protection against diabetes than liraglutide. Thereby, CFP might be an effective strategy for the management and treatment of diabetes.

## Data availability statement

The datasets presented in this study can be found in online repositories. The names of the repository/repositories and accession number(s) can be found below: https://www.ncbi.nlm.nih.gov/, BioProject PRJNA858180.

## Ethics statement

The animal study was reviewed and approved by the Laboratory Animals Ethical Committee of the Zhejiang University of Technology.

## Author contributions

YN: conceptualization, writing-review, editing, project administration, and funding acquisition. AZ: investigation, methodology, writing-original draft, and visualization. YH, NR, SY, SN, LZ, and KZ: data curation and validation. QZ and WL: conceptualization and discussion. TW and ZF: resources, supervision, and funding acquisition. All authors read and approved the final manuscript.

## Conflict of interest

The authors declare that the research was conducted in the absence of any commercial or financial relationships that could be construed as a potential conflict of interest.

## Publisher’s note

All claims expressed in this article are solely those of the authors and do not necessarily represent those of their affiliated organizations, or those of the publisher, the editors and the reviewers. Any product that may be evaluated in this article, or claim that may be made by its manufacturer, is not guaranteed or endorsed by the publisher.
