# Supplementary material for: Compound dietary fiber and high-grade protein diet improves glycemic control and ameliorates diabetes and its comorbidities through remodeling the gut microbiota in mice
Source: Front Nutr. 2022 Jul 22;9:959703. doi: 10.3389/fnut.2022.959703 (PMC9363113; doi:10.3389/fnut.2022.959703)
Supplement: Supplementary file 1 [file Data_Sheet_1.docx]

Supplementary Table 1. Nutrition facts of CFP

| Facts | Per 100g | NRV% | Facts | Per 100g | NRV% |
| --- | --- | --- | --- | --- | --- |
| Calories | 1240KJ | 15% | Vitamin B6 | 1.31mg | 94% |
| Proteins | 19.2g | 32% | Vitamin B12 | 3.92μg | 163% |
| Lipids | 3.6g | 6% | Vitamin C | 170mg | 170% |
| Carbohydrate | 24.5g | 8% | Niacin | 21.21mg | 152% |
| Fibers | 45.5g | 182% | Folic Acid | 259μg DFE | 65% |
| Sodium | 31mg | 2% | Pantothenic Acid | 6.74mg | 135% |
| Vitamin A | 410µg RE | 51% | Phosphorus | 475mg | 68% |
| Vitamin D | 1.7µg | 34% | Magnesium | 132mg | 44% |
| Vitamin E | 16.52mg $\alpha$-TE | 118% | Calcium | 320mg | 40% |
| Vitamin B1 | 1.38mg | 99% | Iron | 9.9mg | 66% |
| Vitamin B2 | 1.51mg | 108% | Zinc | 9.73mg | 65% |

Supplementary Table 2. Primers sequences.

| Name | Forward Primer | Reverse Primer |
| --- | --- | --- |
| *FAS* | 5’-AGAGACGTGTCACTCCTGGACTT-3’ | 5’-GCTGCGGAAACTTCAGAAAAT-3’ |
| *SCD1* | 5’-TGGAGAGCCCCACACACA-3’ | 5’-TGACAGACTGATCGCAGAGAAAG-3’ |
| *Srebp1c* | 5’-GGAGCCATGGATTGCACATT-3’ | 5’-GGCCCGGGAAGTCACTGT-3’ |
| *ACC* | 5’-TGGAGAGCCCCACACACA-3’ | 5’-TGACAGACTGATCGCAGAGAAAG-3’ |
| *Cpt1*$\alpha$ | 5’-AAACCCACCAGGCTACAGTG-3’ | 5’-TCCTTGTAATGTGCGAGCTG-3’ |
| *PPAR*$\alpha$ | 5’-TGGCTGAGAAGACGCTTGTG-3’ | 5’-TTTGCAAAGCCTGGGATAGC-3’ |
| *F4/80* | 5’-CTTTGGCTATGGGCTTCCAGTC-3’ | 5’-GCAAGGAGGACAGAGTTTATCGTG-3’ |
| *IL-1*$\beta$ | 5’-GGGCCTCAAAGGAAAGAATC-3’ | 5’-TACCAGTTGGGGAACTCTGC-3’ |
| *MCP1* | 5’-AGGTCCCTGTCATGCTTCTGG-3’ | 5’-CTGCTGCTGGTGATCCTCTTG-3’ |
| *TNF*$\alpha$ | 5’-ACACCGAGATTTCCTTCAAACTG-3’ | 5’-CCATCTAGGGTTATGATGCTCTTCA-3’ |
| *IL-6* | 5’-CCACTTCACAAGTCGGAGGCTTA-3’ | 5’-GCAAGTGCATCATCGTTGTTCATAC-3’ |
| *Claudin-1* | 5’-CTGGAAGATGATGAGGTGCAGAAGA-3’ | 5’-CCACTAATGTCGCCAGACCTGAA-3’ |
| *ZO-1* | 5’-AAGAATATGGTCTTCGATTGGC-3’ | 5’-ATTTTCTGTCACAGTACCATTTATCTTC-3’ |
| *Occludin* | 5’-GGTTAAAAATGTGTCTGCAGG-3’ | 5’-GGTTAAAAATGTGTCTGCAGG-3’ |
| *MUC-1* | 5’-AGTTACGGTCAGGCTGCTCCGTGGT-3 | 5’-ACCCTCCCGGAAAACCACAGTC-3 |
| *MUC-2* | 5’-GATGGCACCTACCTCGTTGT-3 | 5’-GTCCTGGCACTTGTTGGAAT-3 |
| *Reg3-*$\gamma$ | 5’-ACTCCCTGAAGAATATACCCTCC-3’ | 5’-CGCTATTGAGCACAGATACGAG-3’ |
| $\alpha$*SMA* | 5’-TGTGCTGGACTCTGGAGATG-3’ | 5’-GAAGGAATAGCCACGCTCAG-3’ |
| *Collagen* | 5’-ACGTCCTGGTGAAGTTGGTC-3’ | 5’-CAGGGAAGCCTCTTTCTCCT-3’ |
| *TGF-*$\beta$ | 5’-TGAGTGGCTGTCTTTTGACG-3’ | 5’-TCTCTGTGGAGCTGAAGCAA-3’ |
| *ANP* | 5'-GCTTCCAGGCCATATTGGAG-3' | 5'-GGGGGCATGACCTCATCTT-3' |
| *BNP* | 5'-GAGGTCACTCCTATCCTCTGG-3' | 5'-GCCATTTCCTCCGACTTTTCTC-3' |
| $\alpha$*SA* | 5'-CCCAAAGCTAACCGGGAGAAG-3' | 5'-CCAGAATCCAACACGATGCC-3' |

Supplementary Table 3. Gut microbiota identified in Figure 4A

| OTU | Microbes | OTU | Microbes |
| --- | --- | --- | --- |
| OTU_01 | *Muribaculaceae* | OTU_21 | *Alistipes* |
| OTU_02 | *Allobaculum* | OTU_22 | *Eubacterium coprostanoligenes group* |
| OTU_03 | *Dubosiella* | OTU_23 | *Akkermansia* |
| OTU_04 | *Parasutterella* | OTU_24 | *Clostridia UCG 014* |
| OTU_05 | *Prevotellaceae UCG 001* | OTU_25 | *Lachnoclostridium* |
| OTU_06 | *Parabacteroides* | OTU_26 | *Faecalibaculum* |
| OTU_07 | *Bifidobacterium* | OTU_27 | *Bacteroides* |
| OTU_08 | *Ruminococcaceae UCG 001* | OTU_28 | *Lachnospiraceae NK4A136 group* |
| OTU_09 | *Coriobacteriaceae UCG 002* | OTU_29 | *Staphylococcus* |
| OTU_10 | *Muribaculum* | OTU_30 | *Ruminococcus* |
| OTU_11 | *Mucispirillum* | OTU_31 | *Corynebacterium* |
| OTU_12 | *Alloprevotella* | OTU_32 | *Aerococcus* |
| OTU_13 | *Incertae Sedis* | OTU_33 | *Lactobacillus* |
| OTU_14 | *Desulfovibrio* | OTU_34 | *Clostridia vadinBB60 group* |
| OTU_15 | *Desulfovibrionaceae UCG 001* | OTU_35 | *Lachnospiraceae UCG 002* |
| OTU_16 | *Candidatus Saccharimonas* | OTU_36 | *Lachnospiraceae UCG 001* |
| OTU_17 | *Rikenellaceae RC9 gut group* | OTU_37 | *Clostridium sensu stricto 1* |
| OTU_18 | *Prevotellaceae Ga6A1 group* | OTU_38 | *Oscillospiraceae UCG 002* |
| OTU_19 | *Colidextribacter* | OTU_39 | *Oscillospiraceae UCG 001* |
| OTU_20 | *Helicobacter* | OTU_40 | *Romboutsia* |
