# Supplementary material for: Compound dietary fiber and high-grade protein diet improves glycemic control and ameliorates diabetes and its comorbidities through remodeling the gut microbiota in mice
Source: Front Nutr. 2022 Jul 22;9:959703. doi: 10.3389/fnut.2022.959703 (PMC9363113; doi:10.3389/fnut.2022.959703)
Supplement: Supplementary file 2 [file Data_Sheet_2.docx]

**Figure legends**

**Figure S1.** CFP administration has no effect on pancreatic function. **(A)** Experimental design and schedule of the study. **(B)** Cumulative food intak. **(C)** Immunohistochemistry staining for insulin (red) and glucagon (green) in the pancreas, Scale bar: 100 μm. Data are presented as the means ± SEM, **P* < 0.05, ***P* < 0.01 vs. control; ^#^*P* < 0.05, ^##^*P* < 0.01 vs. Dia.

**Figure S2.** CFP administration reduces the risk of diabetes-related comorbidities. **(A-C)** Effect of CFP on kidney injury. **(A)** H&E, PAS, and Masson staining of kidney sections, Scale bar: 100 µm. **(B)** mRNA expression of *Il-1β*, *Mcp1*, and *Tnf-α* in the kidney. **(C)** mRNA expression of $\alpha$*-Sma*, *Collagen*, and *Tgf-*$\beta$ in the kidney. **(D-E)** Effect of CFP on heart injury. **(D)** mRNA expression of *Il-1β*, *Mcp1*, and *Tnf-α* in the heart. **(E)** mRNA expression of *Anp*, *Bnp*, and $\alpha$*-Sa* in the heart. Data are presented as the means ± SEM, **P* < 0.05, ***P* < 0.01 vs. control; ^#^*P* < 0.05, ^##^*P* < 0.01 vs. Dia.

**Figure S3.** Heatmap of relative abundance of microbiota at the genus level.

**Figure S4.** CFP administration remodels the function of gut microbiota. **(A)** Effects of CFP on the functional profiling of microbial communities. Microbial functional profiling was predicted by PICRUSt and classified by METACYC categories. Significantly changed functions were compared between control and diabetic mice or diabetic and high-dose CFP-treated mice. Data are presented as the means ± SEM. **(B)** Correlation analysis of top 40 genera with functional profiling. Rows correspond to functional profiling shown on the batten, and columns correspond to the specific genus. Colors orange and green denote positive and negative associations, respectively. The intensity of the colors represents the degree of association between the bacterial genus abundances and functional profiling assessed by Pearson correlations. Strats means *P* < 0.05.

**Figure S5.** KEGG pathway enrichment analysis of plasma metabolome. **(A)** KEGG pathway enrichment analysis in diabetic mice versus the control mice. **(B)** KEGG pathway enrichment analysis in high-dose CFP-treated mice versus the diabetic mice.

**Figure S6.** The effects of CFP on arginine metabolism, tyrosine metabolism and glycolysis gene expression in liver. Data are presented as the means ± SEM, **P* < 0.05, ***P* < 0.01 vs. control; ^#^*P* < 0.05, ^##^*P* < 0.01 vs. Dia.

**Figure S7.** Correlation analysis of metabolites with the composition of gut microbiota.
