# Supplementary figures and images for: Compound dietary fiber and high-grade protein diet improves glycemic control and ameliorates diabetes and its comorbidities through remodeling the gut microbiota in mice
Source: Front Nutr. 2022 Jul 22;9:959703. doi: 10.3389/fnut.2022.959703 (PMC9363113; doi:10.3389/fnut.2022.959703)

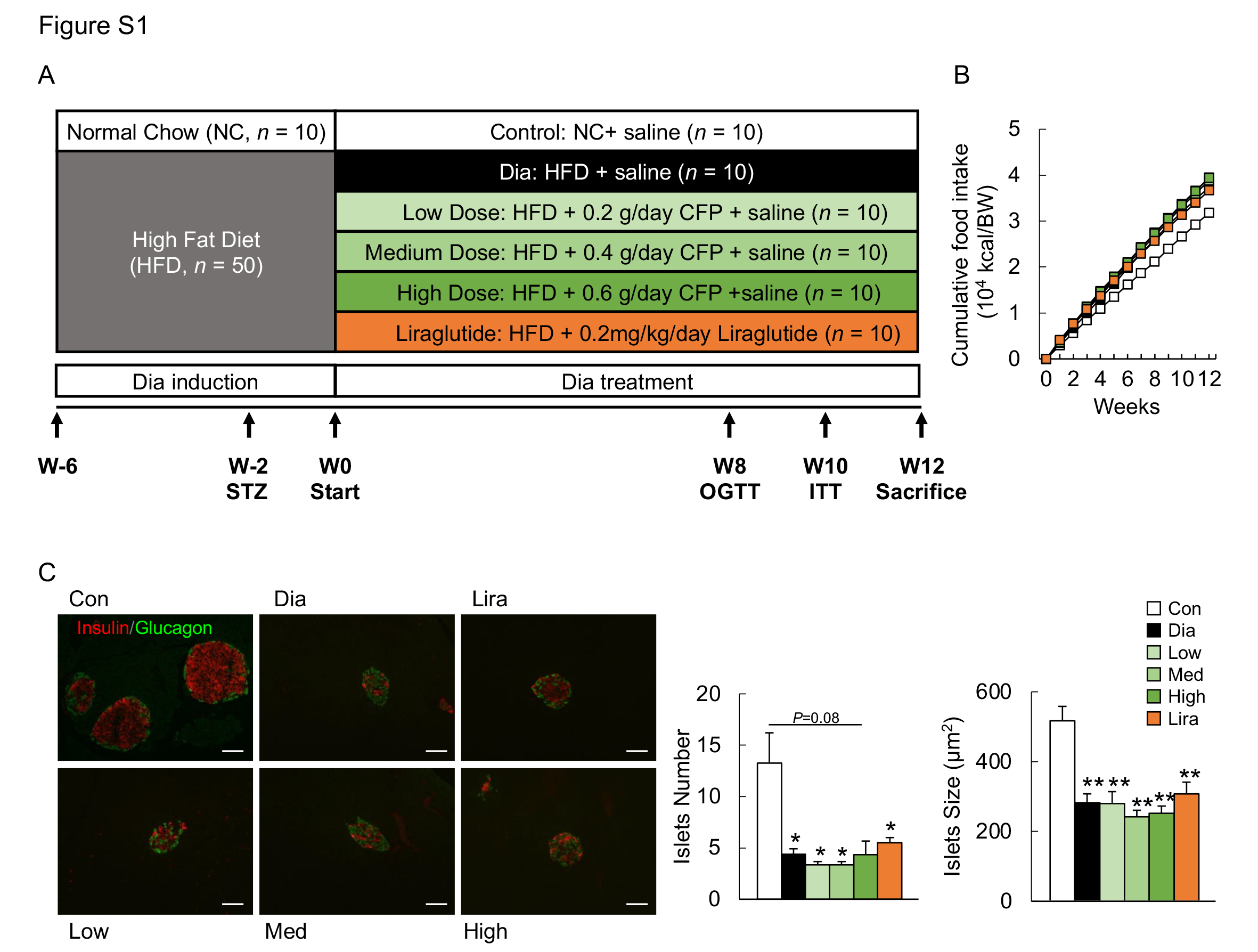

Supplement: Supplementary file 3 [file Image_1.TIF]

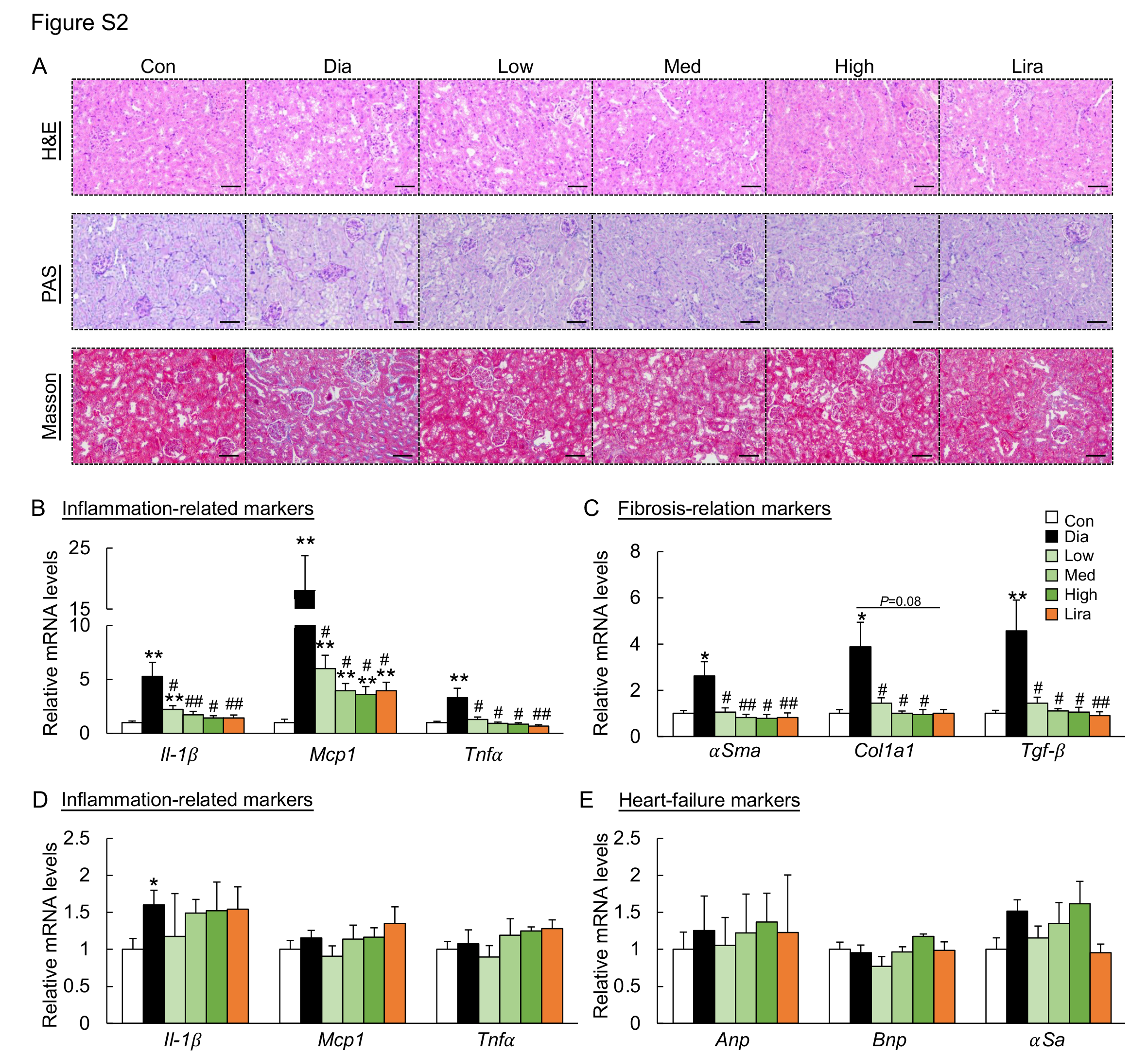

Supplement: Supplementary file 4 [file Image_2.TIF]

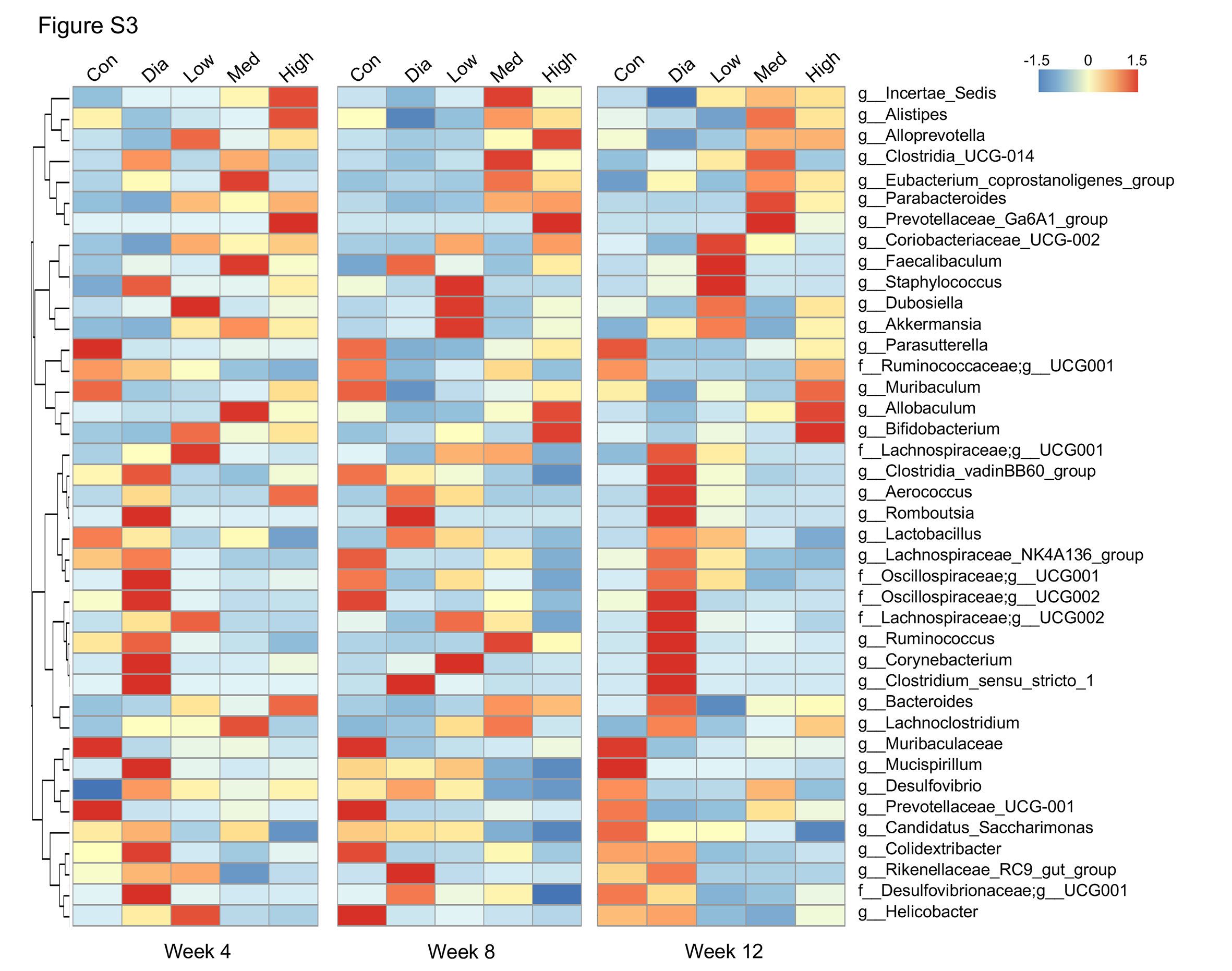

Supplement: Supplementary file 5 [file Image_3.TIF]

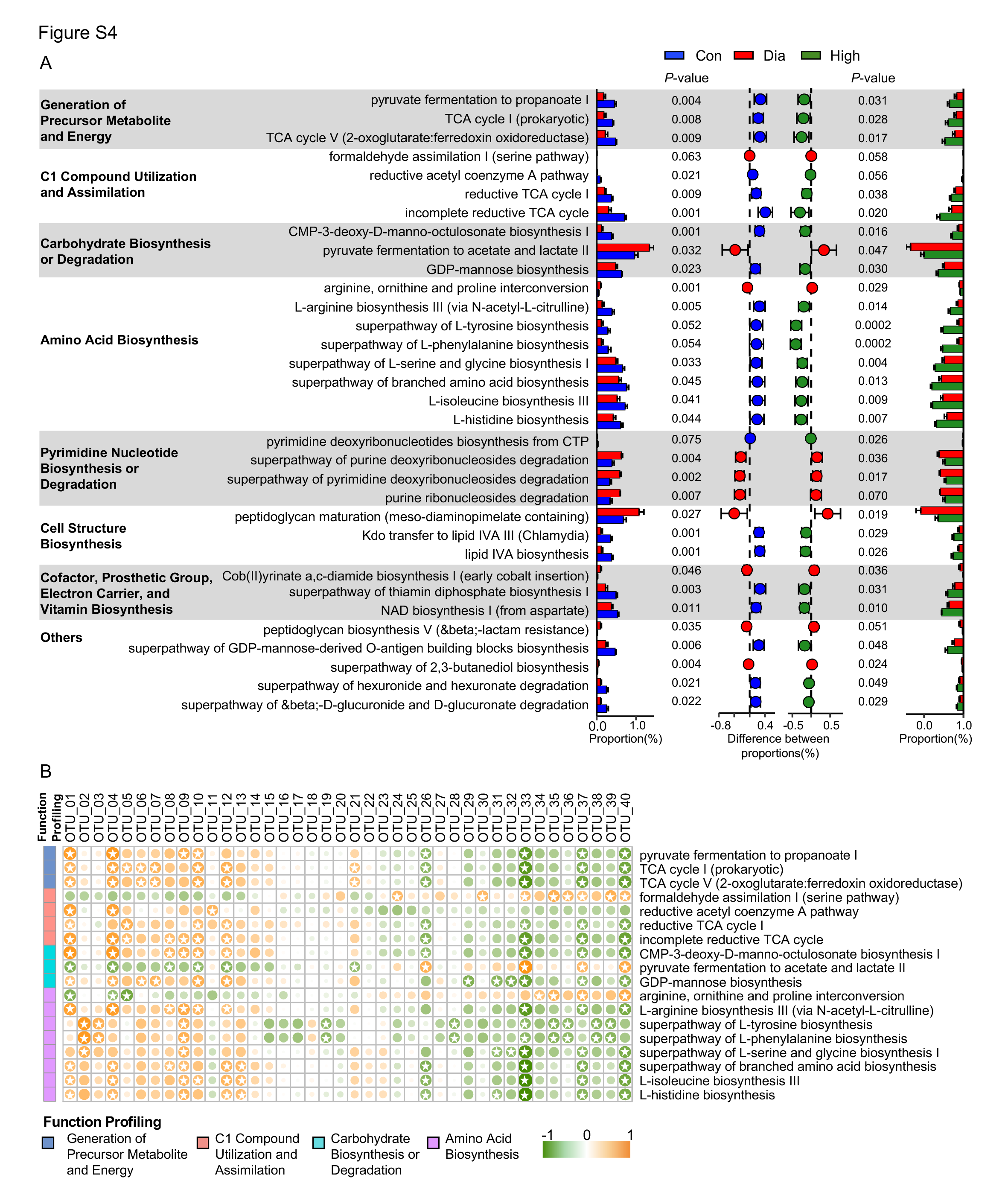

Supplement: Supplementary file 6 [file Image_4.TIF]

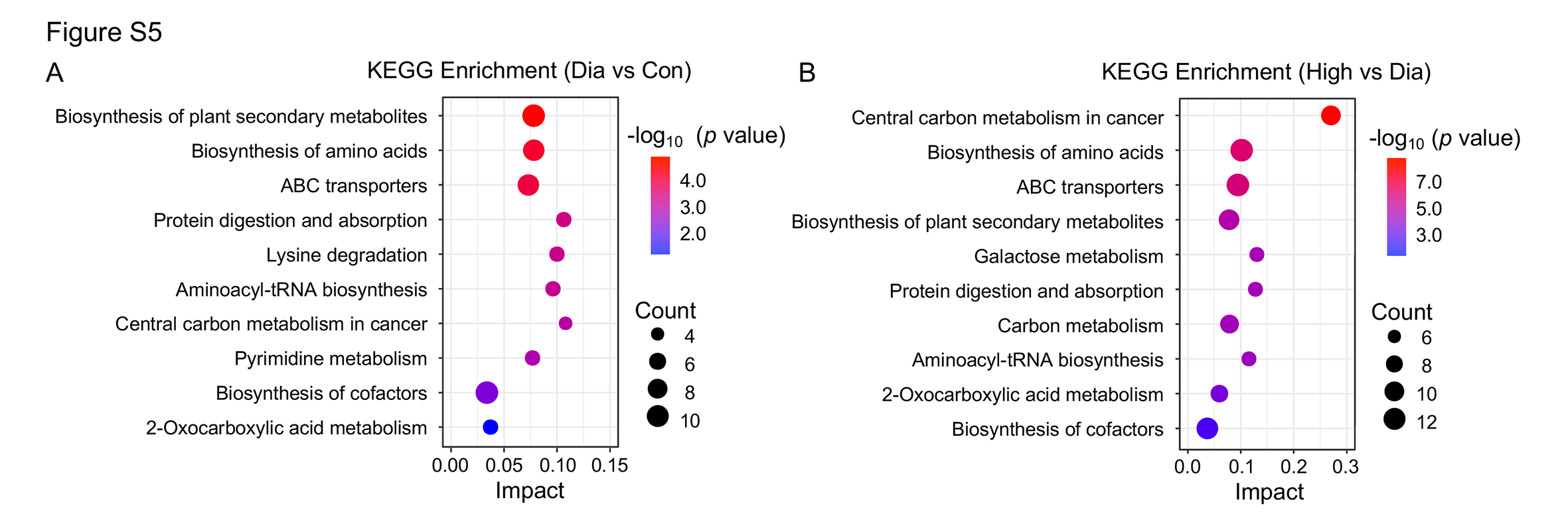

Supplement: Supplementary file 7 [file Image_5.TIF]

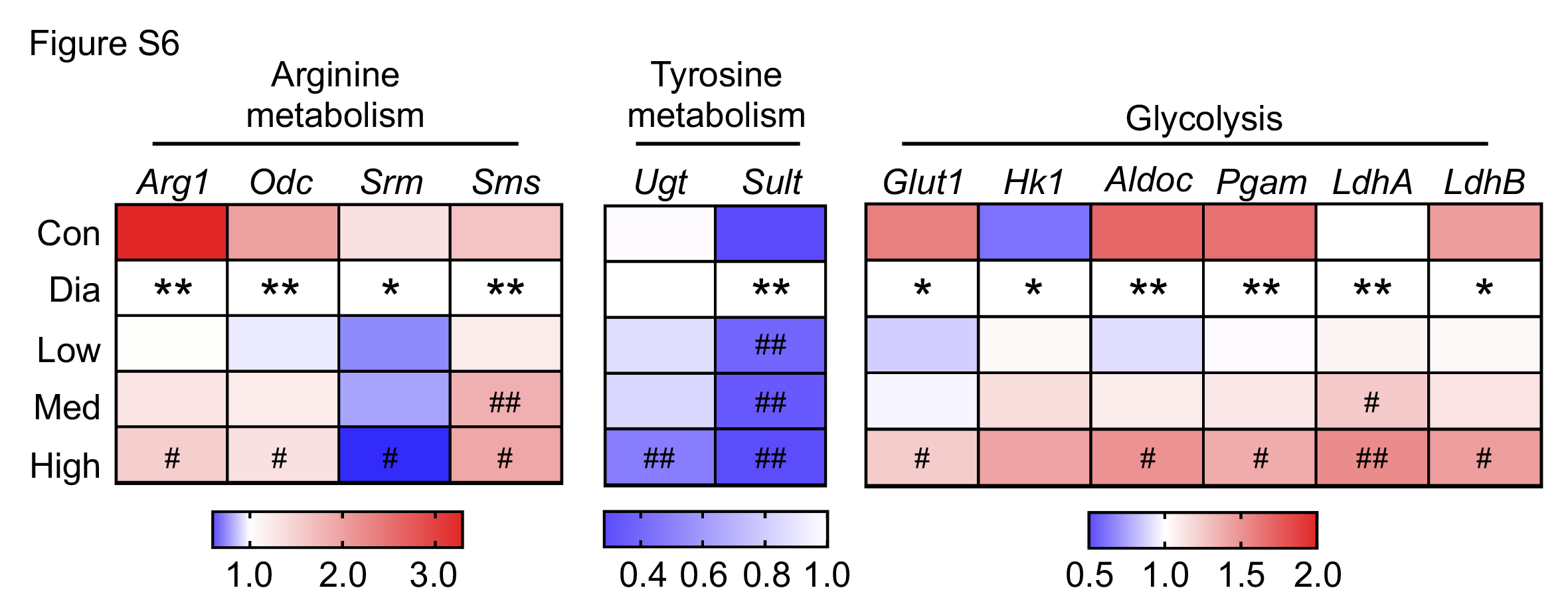

Supplement: Supplementary file 8 [file Image_6.TIF]

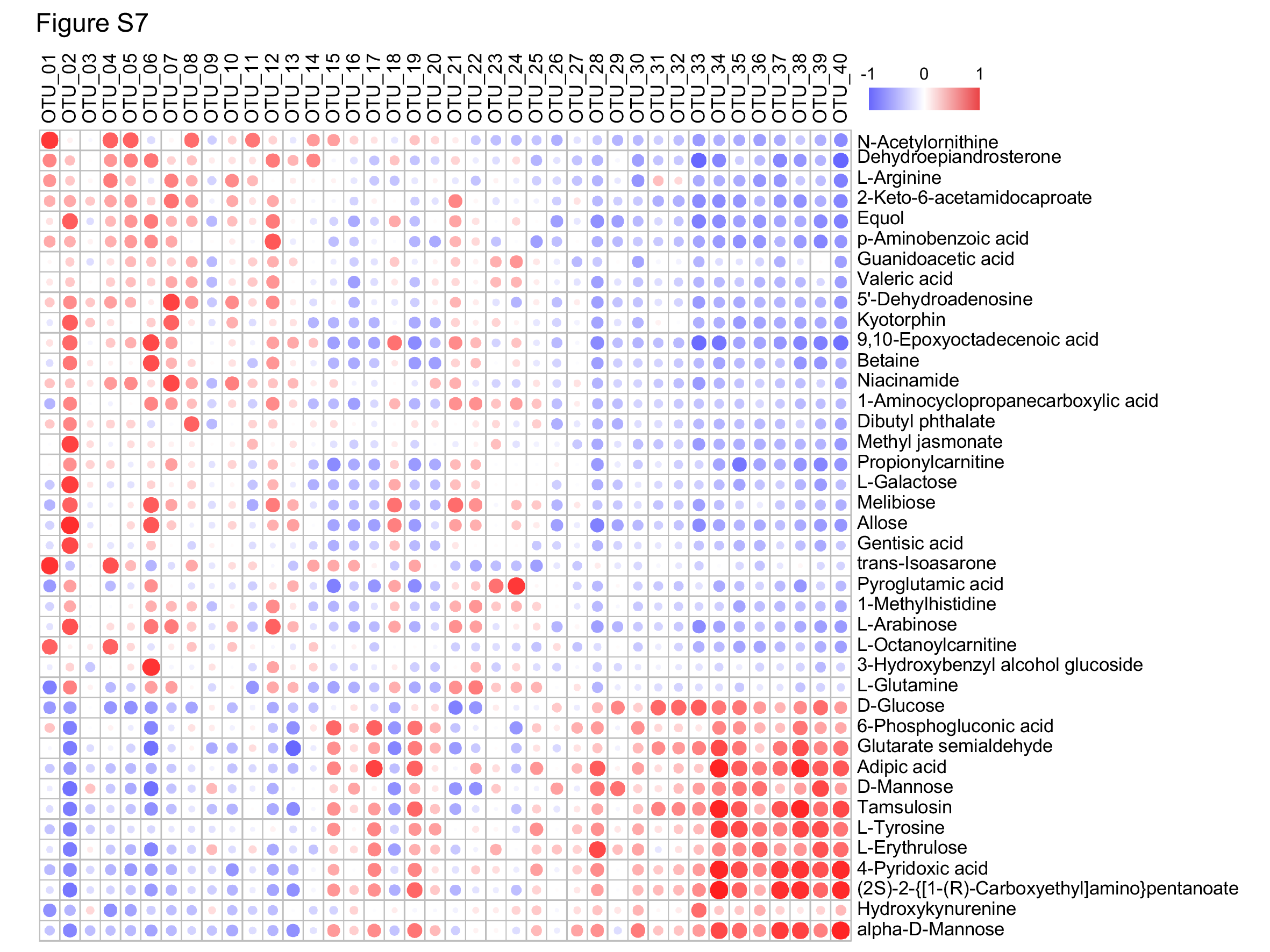

Supplement: Supplementary file 9 [file Image_7.TIF]
